# Spatiotemporal Regularity and Socioeconomic Drivers of the AQI in the Yangtze River Delta of China

**DOI:** 10.3390/ijerph19159017

**Published:** 2022-07-25

**Authors:** Dan Yan, Guoliang Chen, Yu Lei, Qi Zhou, Chengjun Liu, Fan Su

**Affiliations:** 1 School of Public Administration, Zhejiang University of Technology, Hangzhou 310023, China; yandan@zjut.edu.cn (D.Y.); 202001280407@zjut.edu.cn (Y.L.); idealisticbellazhou@outlook.com (Q.Z.); sufan0830@outlook.com (F.S.); 2 Zhejiang Center of Public Opinion and Research, Hangzhou 310023, China; 3 School of Economics, Zhejiang University of Technology, Hangzhou 310023, China; 4 Business School, Zhijiang College of Zhejiang University of Technology, Shaoxing 312030, China; lcjun01@163.com

**Keywords:** AQI, spatiotemporal regularity, Yangtze River Delta, geographically weighted regression, heterogeneity

## Abstract

Air pollution has caused adverse effects on the climate, the ecological environment and human health, and it has become a major challenge facing the world today. The Yangtze River Delta (YRD) is the region with the most developed economy and the most concentrated population in China. Identifying and quantifying the spatiotemporal characteristics and impact mechanism of air quality in this region would help in formulating effective mitigation policies. Using annual data on the air quality index (AQI) of 39 cities in the YRD from 2015 to 2018, the spatiotemporal regularity of the AQI is meticulously uncovered. Furthermore, a geographically weighted regression (GWR) model is used to qualify the geographical heterogeneity of the effect of different socioeconomic variables on the AQI level. The empirical results show that (1) the urban agglomeration in the YRD presents an air pollution pattern of being low in the northwest and high in the southeast. The spatial correlation of the distribution of the AQI level is verified. The spatiotemporal regularity of the “high clustering club” and the “low clustering club” is obvious. (2) Different socioeconomic factors show obvious geographically heterogeneous effects on the AQI level. Among them, the impact intensity of transportation infrastructure is the largest, and the impact intensity of the openness level is the smallest. (3) The upgrading of the industrial structure improves the air quality status in the northwest more than it does in the southeast. The impact of transportation infrastructure on the air pollution of cities in Zhejiang Province is significantly higher than the impact on the air pollution of other cities. The air quality improvement brought by technological innovation decreases from north to south. With the expansion of urban size, there is a law according to which air quality first deteriorates and then improves. Finally, the government should promote the upgrading of key industries, reasonably control the scale of new construction land, and increase the cultivation of local green innovative enterprises.

## 1. Introduction

Air pollution has attracted worldwide attention as both an environmental and public health issue [1]. As the history of the developed countries shows, with the improvement of the economy and industrialization, there have been a lot of air pollution incidents in different countries and regions, causing great economic and health losses. China’s rugged development model in the past few decades has not only promoted great economic and industrial progress but also increased the consumption of various energy resources. The annual emissions of pollutants such as soot and industrial dust can be as high as tens of millions of tons, resulting in a continuous decline in the quality of the atmospheric environment [2]. Frequent smog incidents have aroused people’s concern over air pollution and have caused many social problems in areas such as transportation, production, and health. As an example, direct economic losses from the nationwide heavy haze event in January 2013 reached CNY 23 billion, with 98% of the total loss coming from emergency and outpatient services in health terminals [3]. In addition, smog and sand storms are expanding in an increasing number of areas. There are more deaths each year due to air pollution-related diseases than well-known diseases such as AIDS and breast cancer, and the annual death toll from household and ambient air pollution in China is about 1.2 million to 1.6 million [4]. China has long been concerned with air pollution, enacting numerous regulatory and control measures as a means of fighting it. To improve air quality, China’s Ministry of Ecology and Environment promulgated the new China Air Quality Standard, which is consistent with World Health Organization standards. Furthermore, the State Council of China places great emphasis on the effective mitigation of air pollution and has successively issued policies and regulations such as the Action Plan on the Prevention and Control of Air Pollution and the three-year action plan for cleaner air (the Blue Sky War) [5]. China’s air quality has significantly improved thanks to these air pollution control policies and regulations, but how to balance regional socioeconomic development and air quality is still a complex subject that warrants exploration.

A large number of studies have investigated the regularity of temporal variations and the spatial distribution of different air pollutants. Examples include sulfur dioxide (SO_2_) [6,7,8], particulate matter with a median diameter less than 2.5 µm (PM_2.5_) [9,10,11], and particulate matter with a median diameter less than 10 µm (PM_10_) [12,13]. Research on the spatiotemporal regularity of air pollutants has been carried out extensively in single cities [12,14], urban agglomerations [15], hot regions [16,17,18], and even nationwide [19]. Another kind of research identifies the socioeconomic influencing factors of air pollutants through econometric models. For example, a spatial lag model was used to test the relationship between urbanization and the PM_2.5_ concentration [20]. The spatial autoregressive conditional heteroscedasticity (ARCH) model was adopted to analyze the socioeconomic variation in PM_2.5_ pollution in Chinese cities, and it was found that economic development, the secondary industry, foreign direct investment (FDI), population density, and urbanization affect PM_2.5_ pollution and produce a heterogeneous effect [21]. Similar studies also include Liu et al. (2020b) [22], Qiang et al. (2021) [23], and Halim et al. (2020) [24]. It has also been proven that regional natural conditions affect air pollutant levels [25].

With regional compound air pollution intensifying, China has shifted its focus from mitigating a single pollutant to improving overall air quality [26]. The cause of air pollution is the comprehensive action of multiple pollutants, and a single pollutant cannot properly reflect the broader air quality situation. The air quality index (AQI) is a quantitative measure of air pollution that is dimensionless (Fang et al., 2019). It includes six pollutants: PM_2.5_, PM_10_, SO_2_, nitrogen dioxide (NO_2_), ozone (O_3_), and carbon monoxide (CO).

According to the Technical Regulation on the Ambient Air Quality Index (HJ633-2012) issued in 2012, the AQI value ranges from 0 to 500. As the AQI value increases, the air quality becomes worse. The classification standards for the AQI are shown in Table 1. When the AQI value is 0–50, the air quality is considered “excellent”, which means that there is basically no air pollution. When the AQI value is 51–100, the air quality is considered “good”, which indicates some pollutants may be weakly harmful to the health of very few highly sensitive individuals. When the AQI value is 101–150, the air quality is considered “light pollution”, which means that susceptible people exposed to the air will have mild symptoms and that healthy people will have irritation symptoms. When the AQI value is 151–200, the air quality is considered “moderate pollution”, which indicates that the heart and respiratory systems of healthy people will be affected. When the AQI value is 201–300, the air quality is considered “heavy pollution”, which means that healthy people will begin to have common symptoms. When the AQI value exceeds 300, the air quality is considered “serious pollution”, which means that people’s exercise tolerance will decrease and certain diseases will appear ahead of time.

The majority of severe air pollution in China takes place in urban areas with more developed industries, including Beijing–Tianjin–Hebei and the Yangtze River Delta urban agglomeration. According to the Outline of the Integrated Regional Development of the Yangtze River Delta issued by the State Council of China, the Yangtze River Delta (YRD) urban agglomeration is within the scope of Shanghai, Jiangsu, Zhejiang, and Anhui Provinces and has a total of 41 metropolitan areas. In terms of the country’s comprehensive opening-up pattern, the YRD urban agglomeration plays a pivotal strategic position at the intersection of “the Belt and Road” strategy and the Yangtze River Economic Belt. The YRD is one of the most important engines of economic growth, as well as one of the most important areas for air pollution control, as energy consumption and emissions are on the rise. When compared with a single city, urban agglomerations exhibit a more pronounced contradiction of socioeconomic development and environmental protection, and collaborative government approaches are needed to address air pollution [27]. Studies in the past have focused more on the source analysis, spatial analysis, simulation, and prediction of air pollutants, and few studies have conducted a detailed analysis of the AQI of the YRD. To promote the collaborative efficiency of air pollution governance in various cities of the YRD, there is an urgent need to uncover the spatiotemporal regularity and influencing factors of the AQI in this region to provide a scientific basis for formulating a collaborative governance mechanism for environmental governance.

Based on the issues above, this research makes the following noteworthy contributions: (1) the overall spatiotemporal regularity of the AQI distribution in the YRD is systematically uncovered based on 2015–2018 data covering 39 cities. (2) From the perspective of socioeconomic factors, a more comprehensive, localized, and in-depth understanding of the influence mechanisms of air pollution in the YRD is gained. (3) Additionally, a geographically weighted regression (GWR) model is employed to investigate the substantial spatially heterogeneous effects of socioeconomic factors on varying cities. The remainder of the paper is arranged as follows. The second part introduces the models and data used in this study. Section 3 shows the distribution characteristics of the AQI of the YRD through visualization. In Section 4, the empirical results are presented and discussed. Finally, Section 5 summarizes the main conclusions of this study and provides the corresponding policy implications.

## 2. Methodology and Data

### 2.1. Spatial Autocorrelation Analysis

(1) Global spatial autocorrelation

The first law of geography holds that everything is related to other things, but things that are close to each other will be more closely related [28]. Spatial autocorrelation is a statistical technique for determining the spatial structure of variables, specifically, the spatial correlation between the attribute values of different geographic things distributed in different spatial locations. There is generally a greater correlation between values that are closer together. A positive correlation means that a variable has the same change trend as its adjacent spatial units; that is, there is a phenomenon of spatial agglomeration. A negative correlation means the opposite. 

Global spatial autocorrelation analysis can identify the general pattern of spatial dependence, and global Moran’s I is the most commonly used statistic that can reflect the spatial correlation of an environmental indicator [29]. In this study, this statistic is used to explore the global spatial correlation of the AQI in YRD. The calculation formula is as follows:

(1) $$I=\frac{n}{S_{0}}\times \frac{\sum_{i=1}^{n}\sum_{j=1}^{n}w_{ij}\left(y_{i}-\bar{y}\right)\left(y_{j}-\bar{y}\right)}{\sum_{i=1}^{n}{\left(y_{j}-\bar{y}\right)}^{2}}$$

where $\;S_{0}=\sum_{i=1}^{n}\sum_{j=1}^{n}w_{ij}$, *n* is the total number of spatial elements, $\;y_{i}\;\mathrm{and}\;y_{j}$ represent the attribute values of the *i*-th and *j*-th spatial units, respectively, $\bar{y}$ is the mean value of all spatial unit attribute values, and *w* is the spatial weight matrix. A larger global Moran index represents a higher degree of spatial correlation. The Z score and *p* value are used for statistical tests.

(2) Local spatial autocorrelation

Through a global test of spatial autocorrelation, we can measure whether variables have spatial accumulation globally, but we cannot accurately point out where they form a clustering phenomenon. Local spatial autocorrelation can describe the local association and variations between adjacent units, and explain how the spatial dependence changes with location. The most commonly used indicator is the local Moran’s I. The calculation formula is as follows:

(2) $$I_{i}=\frac{x_{i}-\bar{X}}{S_{i}{}^{2}}\sum_{j=1,j\neq i}^{n}w_{ij}\left(x_{i}-\bar{X}\right)$$

where $S_{i}{}^{2}=\frac{\sum_{j=1,j\neq i}^{n}{\left(x_{j}-\bar{X}\right)}^{2}}{n-1}$, $x_{i}$ is the attribute of element *i*, $\bar{X}$ is the average value of the corresponding attribute, $w_{ij}$ and *n* have the same meaning as above.

### 2.2. Geographically Weighted Regression

In spatial data, the relationship between variables changes due to the change in geographical location. This phenomenon is called spatial nonstationarity or spatial heterogeneity [30]. As the natural resource endowment and socioeconomic development are distributed in an unbalanced manner in the cities of the YRD, there exists interregional spatial correlation or spatial heterogeneity among urban units. Conventional global models (such as linear regression or nonlinear regression) assume that the relationship between variables has spatial homogeneity and only uses one equation to express the relationship between independent variables and dependent variables [31]. The results obtained can explain only the average effect of a variable and cannot properly explain an individual situation [32]. As a result, a model with local variable coefficients can detect spatial nonstationarity, namely, the GWR model, is further proposed based on Foster’s spatially varying parameter regression [33]. The GWR model introduces the geographical location of data into the ordinary linear regression and determines the spatial heterogeneity using the local smoothing processing method to quantitatively provide the spatially varying relationship between dependent variables and multiple independent variables [34,35]. Due to its ability to account for the local effects of spatial objects, it has a more accurate measurement.

The specific expression of the GWR model is as follows:

(3) $$y_{i}=\beta_{0}\left(u_{i},v_{i}\right)+\sum_{k}\beta_{k}\left(u_{i},v_{i}\right)x_{k,i}+\varepsilon_{i}$$

where $\left(u_{i},v_{i}\right)$ is the longitude and latitude of sample *i*, $\beta_{k}\left(u_{i},v_{i}\right)$ is the *k*-th regression coefficient of sample *i*, $y_{i}$ is the dependent variable, and $x_{k,i}$ is the *k*-th independent variable of sample *i*. $\varepsilon_{i}$ is the random error term. The parameter expression of the *i*-th sample is as follows:

(4) $${\hat{\beta }}_{k}\left(u_{i},v_{i}\right)={\left[X^{T}W\left(u_{i},v_{i}\right)X\right]}^{-1}X^{T}W\left(u_{i},v_{i}\right)y\;$$

where W is the spatial weight matrix, and its selection and setting are the core of the GWR model. Based on the distribution characteristics of the value range, kernel functions can be divided into continuous functions (such as the Gaussian function and exponential function) and truncated functions (such as the box-car function, bi-square function, and tri-cube function). In practical applications, the most commonly used are the Gaussian function and bi-square function. After repeated comparison and calculation, the Gaussian function is selected as the most appropriate kernel function of this model.

(5) $$w_{ij}=\exp\left(-d_{ij}^{2}/\theta^{2}\right)\;$$

where $w_{ij}$ is the distance weight from samples *i* and *j*, $d_{ij}$ is the Euclidean distance between samples *i* and *j*, and θ is the bandwidth, which determines the degree of attenuation of the spatial weight with an increase in distance. The greater the bandwidth is, the faster the weight attenuation. Selecting the appropriate bandwidth is an important step in the GWR model [36]. In this study, the Akaike information criterion method is adopted to determine the optimal bandwidth. The smaller AICc is, the more reasonable the bandwidth is [37].

### 2.3. Data Source

The original value of the AQI level came from the Ministry of Ecology and Environment, and the daily air quality detection and monitoring data were published by each province. We averaged the original data to obtain the average annual value of each city. For individual cities, missing data were supplemented through interpolation. Due to the lack of AQI data for Xuancheng and Zhoushan, data on 39 cities in the YRD from 2015 to 2018 were finally taken as the research sample. Moreover, based on previous studies and data availability, five socioeconomic independent variables that may cause urban industrial pollutants were selected: the industrial structure (Ind), transportation (Tran), the openness level (FDI), the scientific and technological innovation level (Inno), and city size (City). The definitions of these factors are shown in Table 2. The socioeconomic data were mainly from the China City Statistical Yearbook.

## 3. Spatiotemporal Regularity of the AQI in the YRD

### 3.1. Spatiotemporal Change in the AQI

**Figure 1:**
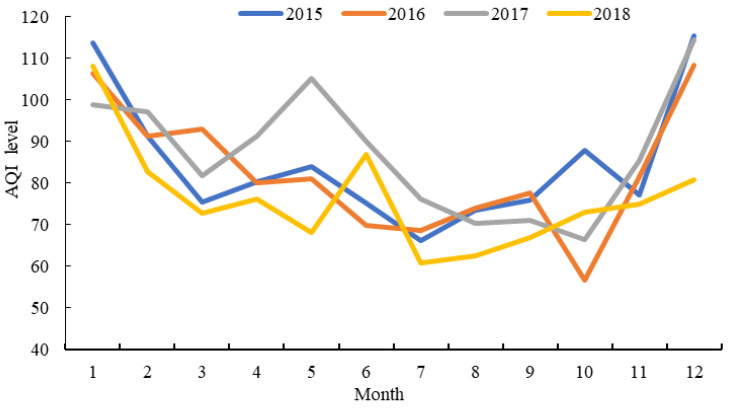
The monthly trend of the AQI value in the YRD from 2015 to 2018.

Based on the monthly value of the AQI in the YRD (see Figure 1), the total trend is a U-shaped curve that is high at both ends and low in the middle. More specifically, the AQI value usually shows a downward trend from January to July, shifts upward in August, and continues to fluctuate until December. The significant seasonal variation may result from the fact that cities consume more energy in winter and have less precipitation and convection than in other seasons. In addition, this research further analyzes the changes in the AQI in different years with variance indicators. The study found that the variance in the AQI in the YRD region was 123.52 in 2015, and the variance expanded to 163.65 in 2017, indicating that the AQI difference between cities in the YRD has an expanding trend. By 2018, the variance in the AQI had dropped to 123.14, and the difference in the AQI between cities had narrowed. We also find that the U-shaped curve has a flattening trend year by year. In terms of the annual trend, the mean AQI value in the YRD decreased by 10.06%, from 84.65 in 2015 to 76.13 in 2018. In terms of interprovincial comparison, the annual mean AQI values from high to low were as follows: Jiangsu Province (93.58), Shanghai (88.5), Anhui Province (79.82), and Zhejiang Province (79.88) in 2015. In 2018, the rankings shifted to Anhui Province (80.08), Jiangsu Province (79.96), Shanghai (70.17), and Zhejiang Province (65.83). The differences among regions slightly expanded.

In addition, we also compare the AQI of the Yangtze River Delta region with the Pearl River Delta region and the Beijing–Tianjin–Tangshan region. The study found that the AQI of the Yangtze River Delta region is higher than that of the Pearl River Delta region, but lower than that of the Beijing–Tianjin–Tangshan region. The average AQI level in the delta region is 58.08, while the average AQI level in the Beijing–Tianjin–Tangshan region is 93.04. Therefore, in terms of environmental quality, the Pearl River Delta region is the best, followed by the Yangtze River Delta region, and the Beijing–Tianjin–Hebei region is the worst. Judging from the changing trend of the AQI, from 2015 to 2018, the AQI of the Pearl River Delta region dropped by 1.63%, while the AQI of the Beijing–Tianjin–Tangshan region dropped by 16.47%, indicating that among these three regions, the Beijing–Tianjin–Tangshan region has the most significant environmental changes.

**Figure 2:**
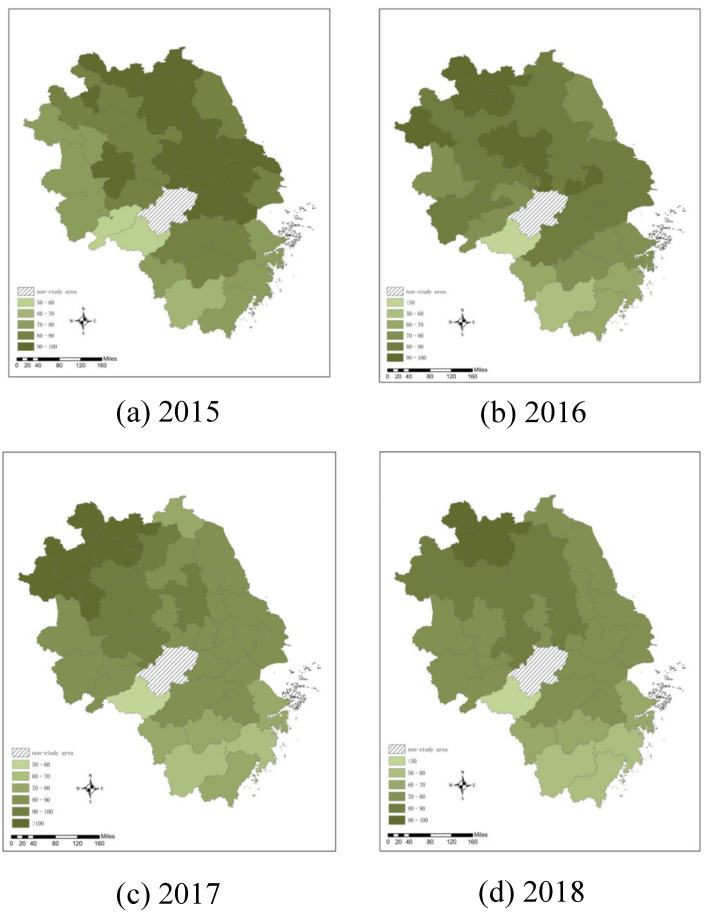
Spatial distribution of the AQI level in the YRD from 2015 to 2018.

In this study, the ArcGIS tool is used to visually display the spatial distribution of the AQI level of the YRD from 2015 to 2018. The results are shown in Figure 2. Different colors represent different AQI levels. The darker the color is, the greater the AQI value, indicating more serious air pollution in a city. Figure 2 shows that most cities in the YRD had AQIs ranging from 50 to 100. Xuzhou (111.67), Huainan (102.92), Fuyang (102.33), Suzhou (108.83), and Bozhou (108.08) showed an excessive AQI in 2017, these cities are facing serious air pollution challenges and tremendous pressure currently, while Huangshan in 2016 (49.92) and in 2018 (42.75) had an AQI less than 50. Overall, the AQI level in Shanghai, Jiangsu Province, and Zhejiang Province showed a downward trend, while the AQI of seven cities in Anhui Province showed an upward trend. The AQI level in the YRD roughly presents a pollution pattern of being high in the northeast and low in the southwest. In 2015, the air pollution in the YRD was mainly concentrated in Jiangsu Province, while there was less air pollution in western Anhui and southern Zhejiang. In 2018, the air pollution in the YRD had shrunk to small areas, such as Huaibei city in northern Anhui Province and Suzhou city and Xuzhou city in Jiangsu Province. This is partly because northern Anhui is an agglomeration area that undertakes industrial transfer from Shanghai, Jiangsu Province, and Zhejiang Province and the production activities are relatively concentrated; thus, the pollution emissions are high. However, through industrial upgrading and optimization, Shanghai, Jiangsu Province, and Zhejiang Province have removed industries with high energy consumption and high emissions from their internal regions; thus, pollutant emissions have been greatly reduced. Meanwhile, the surrounding traffic network will be under greater stress during construction, resulting in greater congestion and traffic inefficiency.

### 3.2. Spatial Trend Distribution of the AQI Level

**Figure 3:**
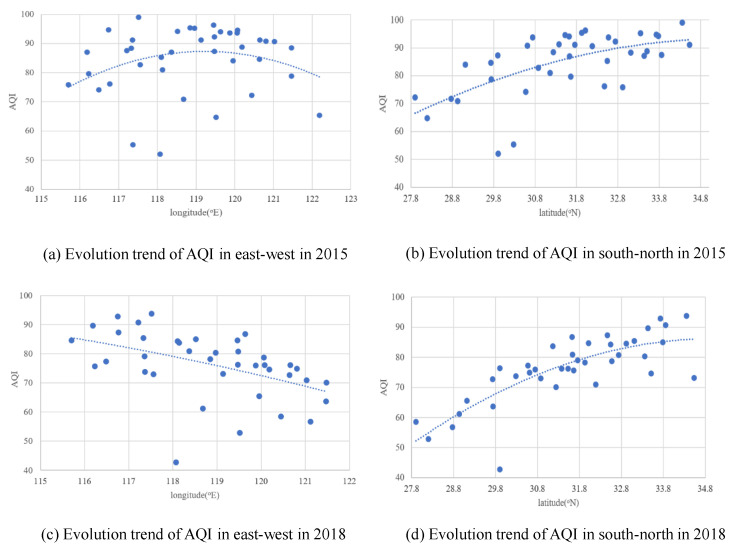
Spatial trend distribution of the AQI in the YRD in 2015 and 2018.

To further investigate the spatiotemporal regularity of the AQI level in the YRD, this study accurately fitted the spatial trend distribution of the AQI value in the east–west and north–south directions of each city in 2015 and 2018 based on the longitude and latitude of each city in the YRD (see Figure 3). In Figure 3, each dot represents a city. Overall, the annual mean AQI values showed a distribution of being high in the north, low in the south, high in the west, and low in the east. Additionally, the fitting curves to longitude and latitude showed varying degrees of uplift in the middle. More details can be found based on the shape and relative position of the fitting curve. In 2015, the annual mean AQI value in the YRD was low in the east and west and high in the midlands, and there was no large gap between the east and west. However, the region’s AQI dropped the most in the east, widening the previous gap between the east and the west in 2018 and showing a distribution of being high in the west and low in the east. In regard to the comparison of longitude, both 2015 and 2018 saw a gradual trend of increasing AQI values from south to north. Furthermore, the slope of the fitting curve in 2018 was greater than that in 2015. This result indicates that the gap between the north and the south was also widening, which, in general, was greater than the gap between the east and the west.

### 3.3. Spatial Autocorrelation Analysis of the AQI

To analyze whether there is spatial correlation of the AQI level in the YRD, Moran’s I of the AQI from 2015 to 2018 is calculated through the spatial weight matrix based on geographical adjacency. In Table 3, the calculation results are shown. Moran’s I shows an increasing trend in general, and the index value increased from 0.5023 in 2015 to 0.5634 in 2018, with both values being significant at the 1% level. Moreover, from 2015–2018, the variance of Moran’s I was only 0.0012, indicating that the deviation of Moran’s I was relatively small. Those results suggest there is not a random distribution of AQI values in the YRD. Instead, the AQI of a city is affected by that of the surrounding areas, and it shows a significantly positive spatial correlation. As a result, the spatial autocorrelation of the AQI in the YRD becomes increasingly significant, presenting a trend of continuous spatial agglomeration.

**Figure 4:**
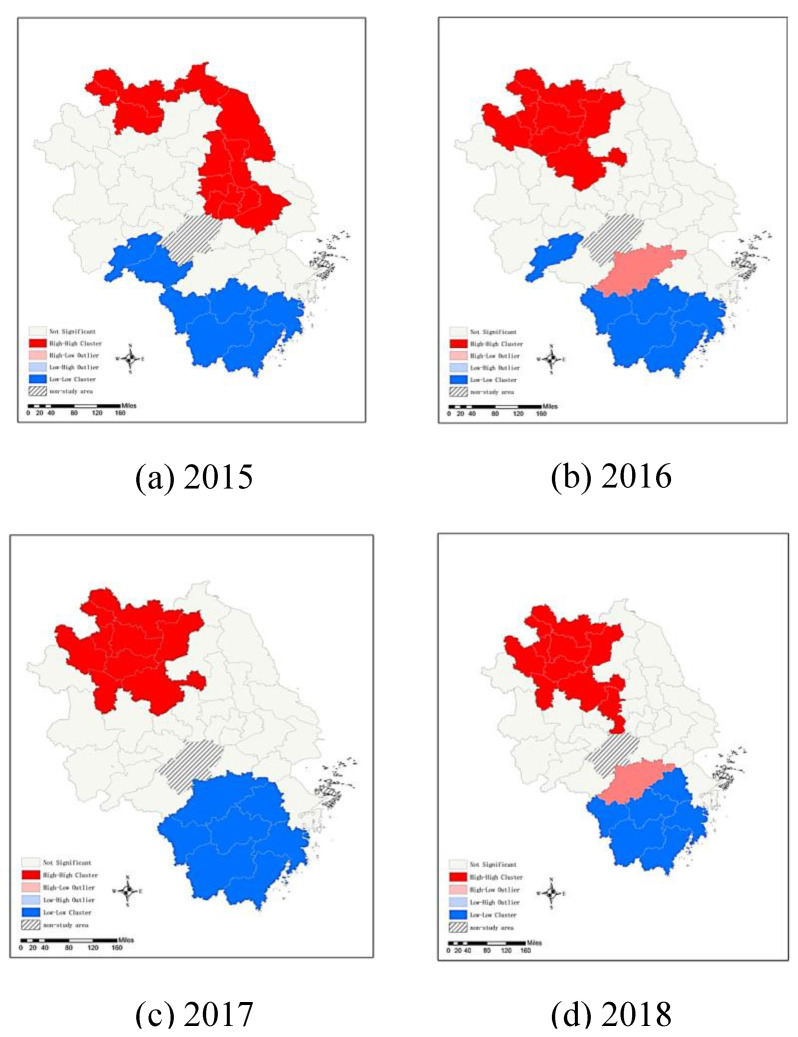
Types of clustering of the AQI level in the YRD from 2015 to 2018.

The results of the global Moran’s I index indicates that the AQI in the YRD presents significant spatial correlation overall but fails to reflect where the agglomeration phenomenon occurs. Further analysis of the spatial characteristics of the AQI is carried out by using the local Moran’s I to determine whether there was local spatial agglomeration. Figure 4 illustrates that the spatial models of the AQI in the YRD can be divided into four types of clustering. High-high (H-H) clustering means that cities with high AQI values are surrounded by cities with high AQI values. The overall AQI values in this region are high, and the degree of spatial variability is small. This is largely due to cities with high AQI and their surrounding areas having similar socioeconomic structures and environmental standards, so these areas more easily form spatially contiguous distribution characteristics. High-low (H-L) clustering means that cities with high AQI values are surrounded by cities with low AQI values. There are high-value outliers and a large degree of spatial variation. The possible reason for this is that areas with a high AQI are more seriously polluted, while the surrounding areas are adjusted by socioeconomic structure to reduce pollution emissions, resulting in a relatively low AQI. Low-low (L-L) clustering means that cities with low AQI values are surrounded by cities with low AQI values. The overall AQI values in this region are low, and the degree of spatial variability is small. This can be explained that, through the transformation and upgrading of the industrial structure, the energy utilization efficiency in the region has been improved, thereby reducing pollution emissions, and through the demonstration effect, the surrounding areas have been driven to reduce pollution emissions, so that there is a spatial interaction effect between low-AQI regions. Low-high (L-H) clustering means that cities with low AQI values are surrounded by cities with high AQI values. There are low-value outliers and a large degree of spatial variation. This can be explained as the transformation of the socioeconomic structure in the region reducing pollution emissions, while the surrounding areas are still developing high-polluting industries, resulting in a higher AQI.

In Figure 4, gray represents the nonsignificant area, red represents H-H clustering, light red represents H-L clustering, light blue represents L-H clustering, and blue represents L-L clustering. The results show that there were 18 cities in 2015 with significant local spatial autocorrelation, including 11 cities showing H-H clustering (61.11%) and seven cities showing L-L clustering (38.89%). In 2018, the number of cities with significant local spatial autocorrelation reached 16, including nine cities showing H-H clustering (56.25%), six cities showing L-L clustering (37.5%), and one city showing H-L clustering (6.25%). In general, the AQI in the YRD illustrates the distribution features of a “high clustering club” and a “low clustering club”.

In 2015, H-H clustering was found in most cities in Jiangsu Province and Suzhou, Anhui Province, whereas L-L clustering was found mostly in Zhejiang Province’s southern cities and Huangshan and Chizhou, Anhui Province. Generalized, the northeast had higher levels of pollution and the southwest had lower levels. However, in 2018, the areas showing H-H clustering shifted from Jiangsu Province to the northern part of Anhui Province, and the areas showing L-L clustering gradually moved from Huangshan and Chizhou in Anhui Province to Zhejiang Province. As a result, the overall pollution pattern in the YRD became high in the northwest and low in the southeast. This change can be explained by the fact that northern Anhui is the main region undertaking the transfer of traditional industries from Shanghai, Jiangsu, and Zhejiang. Furthermore, these industries are relatively polluting and geographically form an industrial agglomeration effect, which makes the AQI distribution in northern Anhui show H-H clustering. However, the Zhejiang government has accelerated industrial transformation and upgrading, especially the deep integration of the digital economy and manufacturing, which has greatly reduced pollutant emissions. Therefore, the air quality has improved not only in Zhejiang but also in surrounding areas.

### 3.4. Hot and Cold Spot Analysis of the AQI

**Figure 5:**
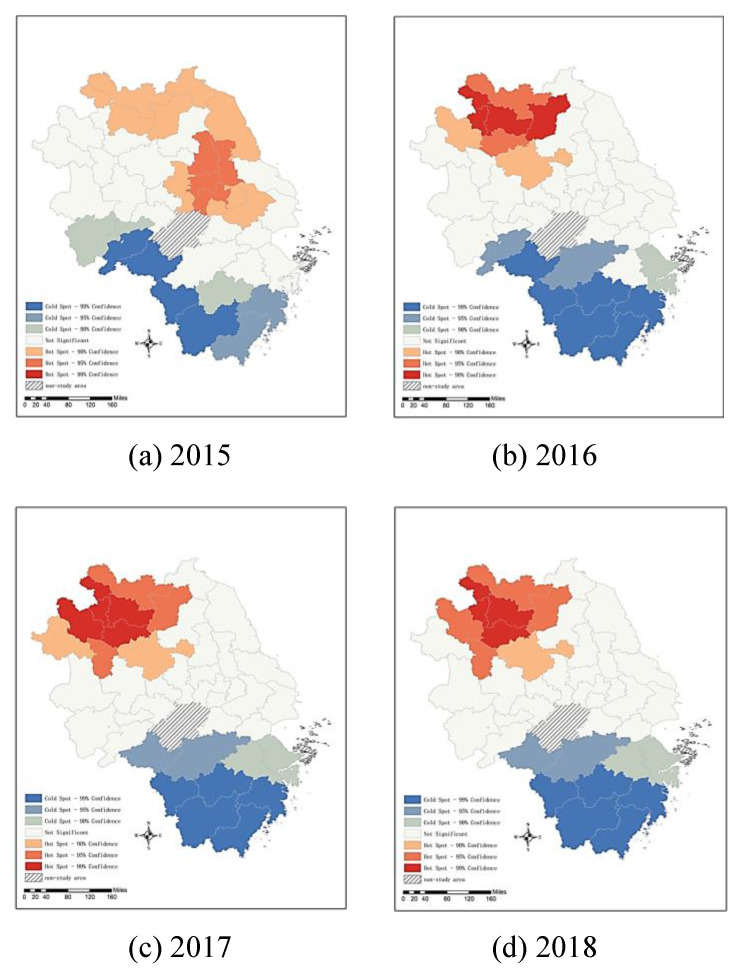
Spatial distribution of hot and cold spots in the YRD from 2015 to 2018.

Hot spot analysis can further detect the key locations of spatial agglomeration and the degree of regional correlation. Additionally, it can determine the contribution of specific regions to global autocorrelation, thus revealing the extent to which Moran’s I masks local instability. The Getis–Ord Gi* statistic can be used to identify significant hot spots (high values) or cold spots (low values). The spatial distribution of hot spots or cold spots is shown in Figure 5.

The results show that from 2015 to 2018, the spatiotemporal evolution of hot spots and cold spots had significant regional characteristics. One characteristic is that hot spots moved westward and northward. In 2015, hot spots were mainly concentrated in southern Jiangsu Province, such as Changzhou, Yangzhou, Zhenjiang, and Taizhou. Later, in 2016, the hot spots shifted to Suqian in Jiangsu Province and Huaibei and Suzhou in Anhui Province. The hot spots further spread to cities in northern Anhui Province, such as Bozhou and Bengbu, in 2017, and by 2018, the hot spots had narrowed to Suzhou, Huaibei and Bengbu.

Another characteristic is that cold spots move eastward and southward. In 2015, cold spots were mainly located in Huangshan, Chizhou in Anhui Province and Quzhou and Lishui in Zhejiang Province. Since then, the cold spots have gradually shifted to southern Zhejiang. By 2018, cold spots were mainly concentrated in southern Zhejiang, such as Quzhou, Lishui, Wenzhou, and Taizhou. This spatiotemporal evolution shows that the AQI is mostly higher in northern Anhui and lower in southern Zhejiang. This fact further demonstrates that the former has relatively serious air pollution, while the latter’s air quality is relatively good. In addition, the difference in air quality between the north and the south is obvious.

## 4. Heterogeneity of Socioeconomic Factors

### 4.1. Model Testing

The above research shows that the AQI in the YRD region has significant spatial heterogeneity. The traditional ordinary least squares (OLS) model ignores the influence of spatial geographical location on the AQI distribution. Therefore, a GWR model is used in this research to empirically identify the regional heterogeneity of influencing factors of AQI in the YRD. Local variations in dependent and independent variables due to location can be effectively dealt with by the GWR model. This research takes the AQI level of 39 cities in the YRD in 2018 as the explained variable, and the relevant indicators from the five dimensions, i.e., the industrial structure (Ind), transportation infrastructure (Tran), the openness level (FDI), the technological innovation level (Inno), and city size (City), are taken as the explanatory variables. ArcGIS 10.7 software is used to carry out regression analysis to explore the factors that might be influencing the differences in the spatial distribution of the AQI. In order to avoid estimation deviations resulting from mutual influence of the indexes, a collinearity test is conducted on the above indexes. The results are shown in Table 4. The variance inflation factor (VIF) of each indicator is less than 10. Therefore, there is no multicollinearity relationship between the indicators selected.

Furthermore, all indicators are standardized. The results show that the GWR model has a higher goodness of fit than the OLS model. Additionally, the significant difference in AIC_C_ values between the two models is greater than 3 (see Table 5), indicating that the GWR model is superior to the OLS model.

### 4.2. Empirical Results

As shown in Table 6, the GWR model calculation results are presented. From high to low, the AQI is influenced by Tran, Ind, Inno, City, and FDI according to the mean of the regression coefficient. Transportation infrastructure has the highest impact on the AQI level, while the openness level has the lowest. In terms of the regression coefficients, there are both positive and negative values, which indicates that the direction and degree of each factor’s influence differ greatly in different cities. Using the traditional regression method, it is possible to obtain the regression coefficient representing only the overall level, while masking some local coefficient characteristics. However, the relationship between the AQI and the influencing factors is not a stable coefficient. As a result of the actual development of different regions, the influencing factors show strong spatial instability; that is, their regression coefficients change significantly with the location. On this basis, ArcGIS software is used to visually analyze the regression coefficients of various factors to create a map of the factors’ spatial distribution.

(1) Industrial Structure

**Figure 6:**
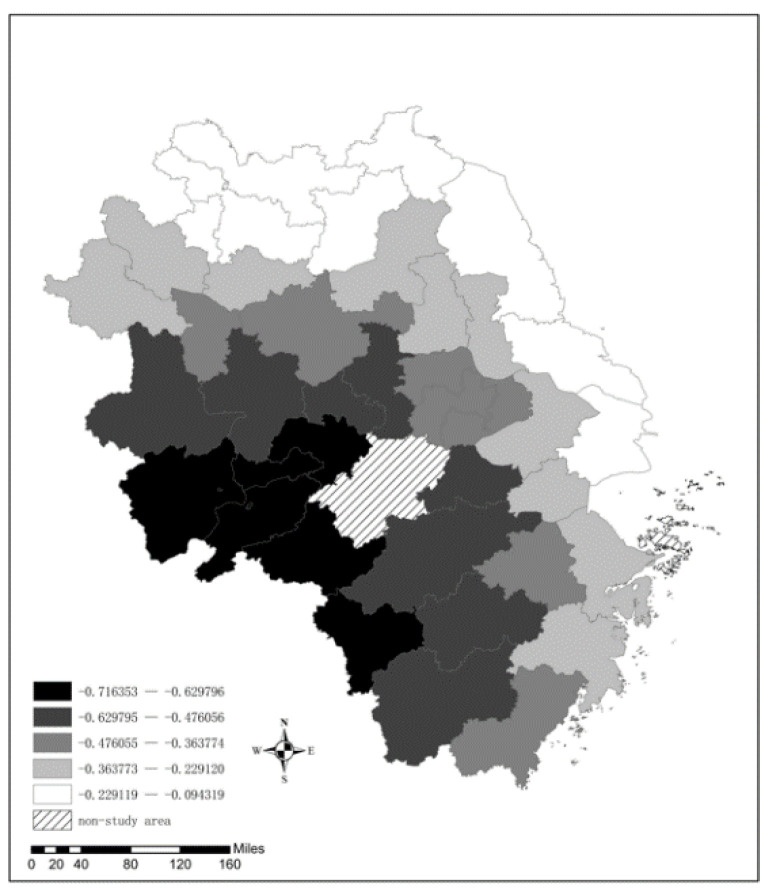
The spatial distribution of the regression coefficient for Ind.

As indicated in Figure 6, a negative correlation exists between upgrading the industrial structure and the AQI level. That is, the higher the proportion of the tertiary industry is, the lower the AQI level, and the better the air quality status. The explanation for this finding lies in the fact that with the increase in the percentage of tertiary industries, the amount of emissions produced by economic activity has decreased and the air quality has improved. Furthermore, east and west of the YRD differ significantly. The negative impact is mainly distributed in the western region, which indicates that the improvement in air quality in the western part of the YRD is significantly higher than that in the eastern part. Moreover, the influence is zonal from west to east, especially in cities such as Wuhu, Tongling, Anqing, Chizhou, Huangshan, and Quzhou. The reason is that the western part of the YRD is mostly located in Anhui Province and the development of the tertiary industry there is relatively insufficient, which makes the marginal promoting effect of industrial upgrading on air quality more obvious. In contrast, the cities in the eastern part of the YRD are mainly located in Zhejiang, Jiangsu, and Shanghai. The tertiary industry in these cities is relatively fully developed; thus, the industrial upgrading of these cities has a comparatively limited effect on the improvement in air quality status. This finding demonstrates the traditional pattern of development of Chinese cities, namely, that when the social development of the city enters industrialization, environmental pollution tends to increase along with GDP. Eventually, however, economic development will reach a point of inflection, environmental pollution will decline with economic development, and environmental quality can coexist harmoniously with economic development.

(2) Transportation Infrastructure

**Figure 7:**
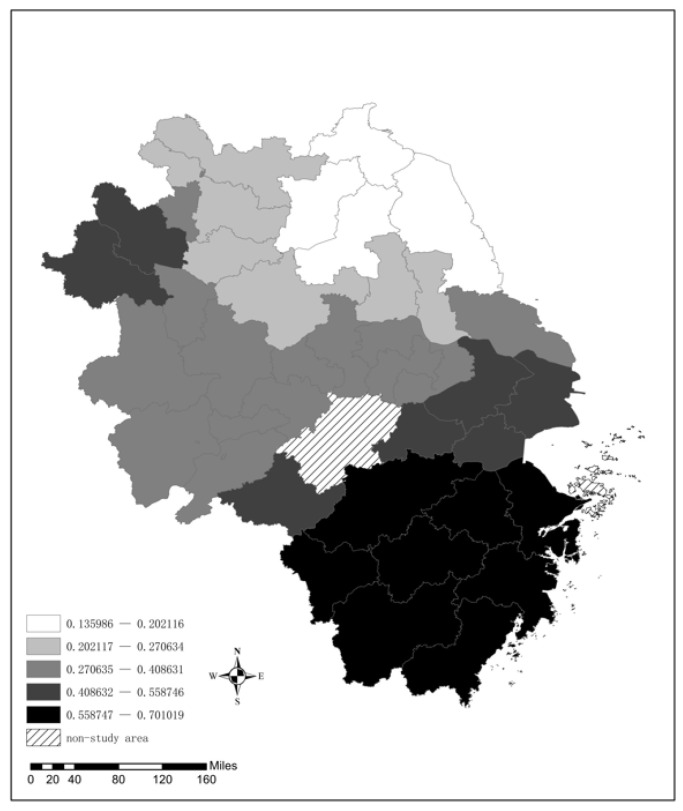
The spatial distribution of the regression coefficient for Tran.

As indicated in Figure 7, a positive correlation exists between transportation infrastructure and the AQI level; that is, the larger the road area is, the worse the air quality status. Furthermore, there is a significant difference between the south and the north in the YRD. The regression coefficient of cities in Zhejiang Province is generally higher than that of other cities in the YRD, and the impact of transportation infrastructure on the AQI in northern Jiangsu and northern Anhui is relatively weak. This finding can be attributed to the strong investment in transportation infrastructure made by the Zhejiang government in recent years. For example, the investment in Zhejiang’s transportation amounted to CNY 373.1 billion in 2018, which was far higher than that of other areas in the YRD. As a result, a large amount of construction dust enters the urban air and aggravates the air pollution. This can also be explained by the phenomena in Jiangsu Province and Anhui Province. The impact of transportation infrastructure on the AQI in southern Jiangsu and southern Anhui is greater than that on their northern counterparts owing to more investment in transportation in southern Jiangsu and southern Anhui.

(3) Openness Level

**Figure 8:**
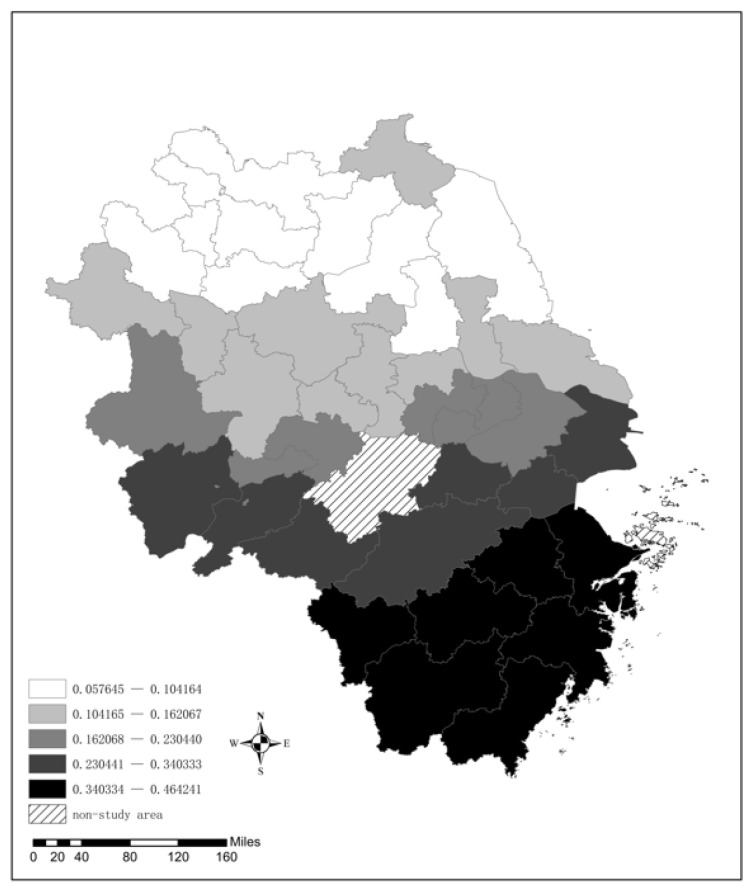
The spatial distribution of the regression coefficient for FDI.

The openness level has a positive correlation effect on the AQI, and the higher the openness level in the YRD is, the worse the air quality. This finding shows that the FDI brought in by economic openness does not bring more advanced technology; rather, it brings more air pollution. This is reasonable for two reasons. First, the governments in the YRD often relax their environmental regulation standards to attract more FDI, thus making the region a “pollution refuge”. In addition, the introduction of FDI has expanded the production scale of the region, increased the emissions of atmospheric particulates to a certain extent, and worsened the air quality. Figure 8 shows that Zhejiang Province is the region with the most serious effect on air pollution due to FDI when considering the spatial distribution of regression coefficients. This result can be explained by the fact that most foreign capital introduced in Zhejiang Province is concentrated in relatively high-pollution industries, such as the textile industry, chemical raw material and chemical product manufacturing industry, and construction industry. Second, compared with southern Jiangsu and western Anhui, the pollution stemming from the introduction of foreign capital in northern Jiangsu and most parts of Anhui is lower. A possible explanation for this finding is the concentration of FDI in Jiangsu’s south, which makes air pollution from foreign investment in these areas more severe than in northern Jiangsu. However, although Anhui does not attract a large amount of foreign capital because of its locational disadvantage, the fragile ecological environment in western Anhui makes the air pollution stemming from foreign investment stronger than that in other parts of Anhui.

(4) Technological Innovation Level

**Figure 9:**
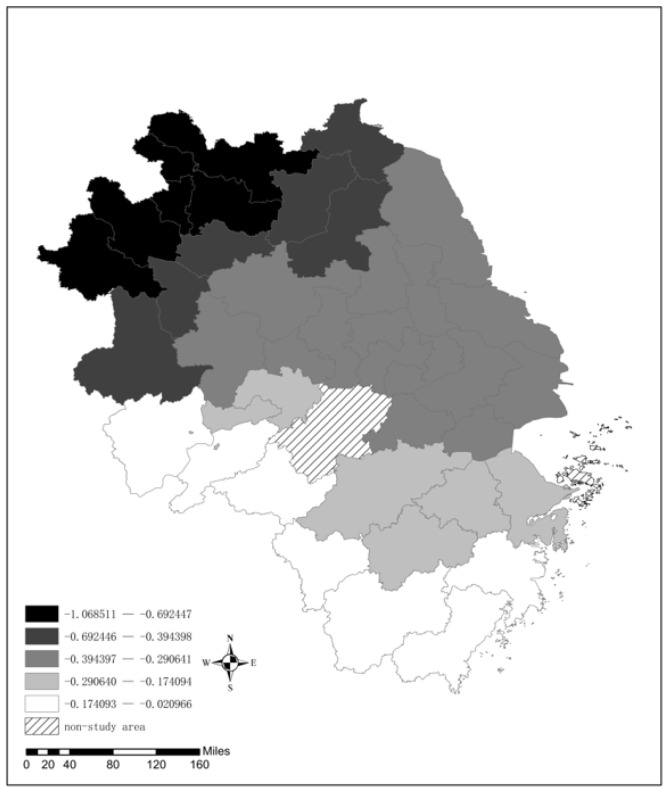
The spatial distribution of the regression coefficient for Inno.

A negative correlation exists between the technological innovation level and the AQI in the YRD (see Figure 9). Higher levels of scientific and technological innovation lead to better air quality. This result shows that a stronger research capacity allows cities to conduct high-tech research that supports industrial upgrading and productivity increases. Scientific and technological innovation has strengthened the application of emerging technologies in the production process and the clean production mode has been gradually promoted [38]. As a result, the emission of air pollutants in the production process has been reduced, thus improving the air quality. The spatial distribution of the regression coefficient indicates that scientific and technological innovation has a decreasing effect on air quality from north to south, especially in northern Anhui and northern Jiangsu, and technological innovation’s effect on air quality is the most obvious. It is because these areas have relatively low levels of original scientific and technological innovation and air quality is more sensitive to changes in scientific and technological innovation. In contrast, the high level of scientific and technological innovation in southern Jiangsu, Shanghai, and northern Zhejiang makes the improvement effect relatively limited.

(5) City Size

**Figure 10:**
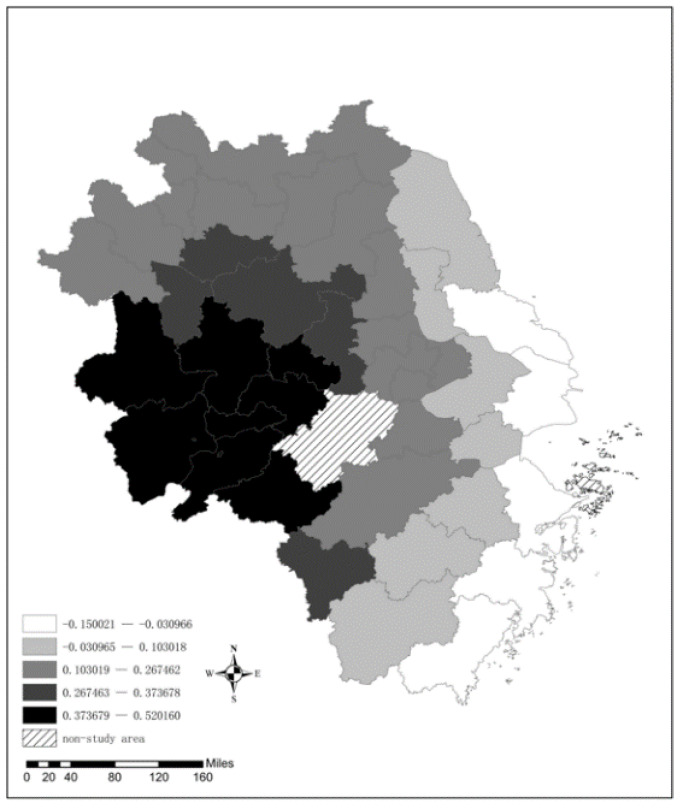
The spatial distribution of the regression coefficient for City.

There are two different effects of city size on the AQI in the YRD. Thirty-four cities have a positive correlation effect, while the remaining five cities have a negative correlation effect. This result can be explained by the environmental Kuznets curve. Typically, smaller cities tend to have low levels of economic development, and the degree of environmental pollution is also lower; however, as the city size expands, the development of urban construction is rapid, and a large amount of construction dust enters the urban air, aggravating urban air pollution. From the spatial distribution of the regression coefficient (see Figure 10), the expansion of cities in western Anhui has the most significant effect on air quality deterioration, mainly due to the good ecological environment of these cities; additionally, these cities have some room for improvement when it comes to industrial structure and infrastructure development, the boom in urban construction easily causes air pollution. Moreover, the effect of city size on the spatial distribution of pollution decreases from west to east, especially in Shanghai, Nantong, Ningbo, Taizhou, and Wenzhou in the east, and the expansion of the size of cities has improved the air quality there. That is because these cities have more environmentally friendly production lifestyles and infrastructure, and can allocate and utilize resources more effectively.

## 5. Conclusions

Since the reform and opening up, the YRD has become the center undertaking global industrial transfer, indirectly changing the pattern of pollution emissions of the region. By using the spatial autocorrelation test and GWR model, this study explores the evolution of the spatiotemporal regularity and impact mechanism of the AQI level in 39 cities of the YRD from 2015 to 2018. Doing so holds great significance for jointly controlling high-energy consumption and high-emission industries, realizing coordinated governance among cities, and building a world-class green development urban agglomeration with global influence. **The main conclusions of this study are as follows**. From the perspective of the monthly variation, the average monthly AQI level of the urban agglomeration in the YRD roughly presents a U-shaped curve in the twelve months of the year. From the perspective of geographical distribution, the urban agglomeration in the YRD presents an air pollution pattern of being low in the northwest and high in the southeast. The most polluted areas are concentrated in Huaibei, Suzhou, and Xuzhou. Furthermore, the spatial autocorrelation of the AQI level is verified, and the distribution regularity of the “high clustering club” and the “low clustering club” is obvious.

More importantly, the socioeconomic development of this urban agglomeration has a heterogeneous impact on its air quality. The impact intensity of transportation infrastructure is the largest, and the impact intensity of the openness level is the smallest. The upgrading of the industrial structure improves the air quality status in the northwest more than in the southeast. The impact of transportation infrastructure on the air pollution of cities in Zhejiang Province is significantly higher than that of other cities. The air pollution caused by the introduction of foreign capital is more obvious in Zhejiang Province, and the air quality improvement brought by technological innovation decreases from north to south. At the same time, with the expansion of the size of cities, there is a law according to which air quality first deteriorates and then improves.

The coordinated development of the YRD needs to take the urban agglomeration as the main body to realize overall green development. According to the above empirical results, and the corresponding discussion, the following policy implications for the improvement of air quality status in the YRD are drawn.

First, the government should raise the elimination standard for the backward production capacity in the YRD and promote the upgrading of key industries. Specifically, it can be promoted in the following ways: strictly implementing unified special emission limits on air pollutants; promoting the transformation of the ultralow emissions of coal-fired power units; and carrying out rectification within a time limit for key industries such as steel, cement, and flat glass. In addition, volatile organic compound (VOC) pollution in key industries, such as the petrochemical, coating, packaging, and printing industries, should be treated. Through relevant measures, the rationalization, advancement, and efficiency of the industrial structure can be gradually realized.

Second, the grid spatial pattern suitable for the resource and environmental carrying capacity should be reasonably constructed. The areas with phased saturation of the resource and environmental carrying capacity are mainly distributed in Shanghai and southern Jiangsu and around Hangzhou Bay. The scale and opening intensity of new construction land in these cities should be strictly controlled. Areas with great potential for the resource and environmental carrying capacity are mainly distributed in central Jiangsu, central Zhejiang, central Anhui, and some coastal areas, and the industrial space of these areas can be appropriately expanded. At the same time, the government can also learn from the public management experience of the world’s advanced urban agglomerations and actively promote the application of new energy and related infrastructure construction, thus improving the urban carrying capacity.

Finally, the government should strengthen the cultivation of local green innovative enterprises, guide foreign capital to be invested more in the service industry, and improve the quality and level of foreign capital utilization to avoid becoming a place to which global high-pollution industries are transferred. Green transformation is inseparable from scientific and technological innovation. The government should pay attention to increasing the investment in green innovation, give full play to the radiation and driving role of Shanghai as a core city, and drive the overall local technological and ecological construction [39,40]. Notably, it should also give full play to the comparative advantages of various cities and coordinate the relationship between coastal cities and hinterland cities, regional central cities, and small or medium-sized cities.

## Figures and Tables

**Table 1:** Classification standards for the AQI.

AQI Value	Classification	Air Quality Level
0~50	Level 1	Excellent
51~100	Level 2	Good
101~150	Level 3	Light pollution
151~200	Level 4	Moderate pollution
201~300	Level 5	Heavy pollution
>300	Level 6	Serious pollution

**Table 2:** Definitions of the socioeconomic variables.

Abbreviation	Variables
Ind	The ratio of the output value of the tertiary industry to that of the secondary industry
Tran	Road area
Inno	The number of patents authorized
City	Population
FDI	The actual use of foreign capital as a percentage of GDP

**Table 3:** Moran’s I of the AQI in the YRD, 2015–2018.

Year	Moran’s I	Expectation Index	Z Score	*p* Value
2015	0.5061	−0.0263	5.5495	0.00
2016	0.4913	−0.0263	5.4248	0.00
2017	0.5496	−0.0263	5.9235	0.00
2018	0.5634	−0.0263	6.1176	0.00

**Table 4:** Descriptive statistics of the explanatory variables.

Variable	Mean	Std. Dev	Minimum	Maximum	VIF
Ind	1.1027	0.3278	0.6465	2.3472	1.76
Tran	3661.615	3357.416	652	15,904	3.05
Inno	19,453.79	20,976.73	1097	92,460	3.12
FDI	0.0244	0.0173	0.0033	0.0855	1.03
City	537	274.38	149	1459	1.65

**Table 5:** Parameter estimation and test results of the GWR model.

Parameter	Value
Bandwidth	2.462822
Residual Squares	8.343186
Effective Number	15.666475
Sigma	0.597965
AICc	93.114997
R^2^	0.786072
Adjusted R^2^	0.651606

**Table 6:** Calculation results of the GWR model.

Variable	Minimum	Maximum	Mean	Median	Std. Dev
Ind	−0.7164	−0.0943	−0.3899	−0.3699	0.1805
Tran	0.136	0.701	0.4042	0.3699	0.1561
Inno	−1.0685	−0.021	−0.3548	−0.3065	0.2233
FDI	0.0576	0.4642	0.2129	0.1754	0.1241
City	−0.15	0.5202	0.2245	0.2321	0.1814

## Data Availability

Due to the confidentiality and privacy of the data, they will only be provided upon reasonable request.

## References

[1] Jiang W., Gao W., Gao X., Ma M., Zhou M., Du K., Ma X. (2021). Spatio-temporal heterogeneity of air pollution and its key influencing factors in the Yellow River Economic Belt of China from 2014 to 2019. J. Environ. Manag..

[2] Zhang Y.L., Cao F. (2015). Fine particulate matter (PM_2.5_) in China at a city level. Sci. Rep..

[3] Mu Q., Zhang S. (2013). An evaluation of the economic loss due to the heavy haze during January 2013 in China. China Environ. Sci..

[4] World Health Organization (2021). World Health Statistics 2021: Monitoring Health for the SDGs, Sustainable Development Goals.

[5] Zeng Y., Cao Y., Qiao X., Seyler B.C., Tang Y. (2019). Air pollution reduction in China: Recent success but great challenge for the future. Sci. Total Environ..

[6] Zhang H., Di B., Liu D., Li J., Zhan Y. (2019). Spatiotemporal distributions of ambient SO_2_ across China based on satellite retrievals and ground observations: Substantial decrease in human exposure during 2013–2016. Environ. Res..

[7] Jiang L., Zhou H., He S. (2020). The role of governments in mitigating SO_2_ pollution in China: A perspective of fiscal expenditure. Environ. Sci. Pollut. Res..

[8] Mao M., Sun H., Zhang X. (2020). Air pollution characteristics and health risks in the Yangtze river economic belt, China during winter. Int. J. Environ. Res. Public Health.

[9] Han L., Zhou W., Li W., Li L. (2014). Impact of urbanization level on urban air quality: A case of fine particles (PM_2.5_) in Chinese cities. Environ. Pollut..

[10] Yan D., Lei Y., Shi Y., Zhu Q., Li L., Zhang Z. (2018). Evolution of the spatiotemporal pattern of PM_2.5_ concentrations in China–A case study from the Beijing-Tianjin-Hebei region. Atmos. Environ..

[11] Liu X.J., Xia S.Y., Yang Y., Wu J.F., Zhou Y.N., Ren Y.W. (2020). Spatiotemporal dynamics and impacts of socioeconomic and natural conditions on PM_2.5_ in the Yangtze River Economic Belt. Environ. Pollut..

[12] Xu L., Batterman S., Chen F., Li J., Zhong X., Feng Y., Rao Q., Chen F. (2017). Spatiotemporal characteristics of PM_2.5_ and PM_10_ at urban and corresponding background sites in 23 cities in China. Sci. Total Environ..

[13] Liu X., Hadiatullah H., Tai P., Xu Y., Zhang X., Schnelle-Kreis J., Schloter-Hai B., Zimmermann R. (2021). Air pollution in Germany: Spatio-temporal variations and their driving factors based on continuous data from 2008 to 2018. Environ. Pollut..

[14] Yu S., Yin S., Zhang R., Wang L., Su F., Zhang Y., Yang J. (2020). Spatiotemporal characterization and regional contributions of O_3_ and NO_2_: An investigation of two years of monitoring data in Henan, China. J. Environ. Sci..

[15] Miao L., Liu C., Yang X., Kwan M.-P., Zhang K. (2022). Spatiotemporal heterogeneity analysis of air quality in the Yangtze River Delta, China. Sustain. Cities Soc..

[16] Zhou W., Chen C., Lei L., Fu P., Sun Y. (2021). Temporal variations and spatial distributions of gaseous and particulate air pollutants and their health risks during 2015–2019 in China. Environ. Pollut..

[17] Fang X., Fan Q., Liao Z., Xie J., Xu X., Fan S. (2019). Spatial-temporal characteristics of the air quality in the Guangdong–Hong Kong–Macau Greater Bay Area of China during 2015–2017. Atmos. Environ..

[18] Qin S., Liu F., Wang C., Song Y., Qu J. (2015). Spatial-temporal analysis and projection of extreme particulate matter (PM_10_ and PM_2.5_) levels using association rules: A case study of the Jing-Jin-Ji region, China. Atmos. Environ..

[19] Sun Z., Zhan D., Jin F. (2019). Spatio-temporal characteristics and geographical determinants of air quality in cities at the prefecture level and above in China. Chin. Geogr. Sci..

[20] Zhang X., Gu X., Cheng C., Yang D. (2020). Spatiotemporal heterogeneity of PM_2.5_ and its relationship with urbanization in North China from 2000 to 2017. Sci. Total Environ..

[21] Yan D., Ren X., Zhang W., Li Y., Miao Y. (2022). Exploring the real contribution of socioeconomic variation to urban PM_2.5_ pollution: New evidence from spatial heteroscedasticity. Sci. Total Environ..

[22] Liu X., Zou B., Feng H., Liu N., Zhang H. (2020). Anthropogenic factors of PM_2.5_ distributions in China’s major urban agglomerations: A spatial-temporal analysis. J. Clean. Prod..

[23] Qiang W., Lin Z., Zhu P., Wu K., Lee H.F. (2021). Shrinking cities, urban expansion, and air pollution in China: A spatial econometric analysis. J. Clean. Prod..

[24] Halim N.D.A., Latif M.T., Mohamed A.F., Maulud K.N.A., Idrus S., Azhari A., Othman M., Sofwan N.M. (2020). Spatial assessment of land use impact on air quality in mega urban regions, Malaysia. Sustain. Cities Soc..

[25] Liu H., Fang C., Zhang X., Wang Z., Bao C., Li F. (2017). The effect of natural and anthropogenic factors on haze pollution in Chinese cities: A spatial econometrics approach. J. Clean. Prod..

[26] Feng Y., Ning M., Lei Y., Sun Y., Liu W., Wang J. (2019). Defending blue sky in China: Effectiveness of the “Air Pollution Prevention and Control Action Plan” on air quality improvements from 2013 to 2017. J. Environ. Manag..

[27] Feng T., Du H., Lin Z., Zuo J. (2020). Spatial spillover effects of environmental regulations on air pollution: Evidence from urban agglomerations in China. J. Environ. Manag..

[28] Tobler W.R. (1970). A computer movie simulating urban growth in the Detroit region. Econ. Geogr..

[29] Moran P.A.P. (1948). The interpretation of statistical maps. J. R. Stat. Soc. Ser. B Methodol..

[30] Yang X., Wang S., Zhang W., Zhan D., Li J. (2017). The impact of anthropogenic emissions and meteorological conditions on the spatial variation of ambient SO_2_ concentrations: A panel study of 113 Chinese cities. Sci. Total Environ..

[31] Gilbert A., Chakraborty J. (2011). Using geographically weighted regression for environmental justice analysis: Cumulative cancer risks from air toxics in Florida. Soc. Sci. Res..

[32] Wang J., Wang S., Li S. (2019). Examining the spatially varying effects of factors on PM_2.5_ concentrations in Chinese cities using geographically weighted regression modeling. Environ. Pollut..

[33] Fotheringham A.S., Charlton M.E., Brunsdon C. (2001). Spatial variations in school performance: A local analysis using geographically weighted regression. Geogr. Environ. Model..

[34] Mennis J. (2006). Mapping the results of geographically weighted regression. Cartogr. J..

[35] Guo B., Wang X., Pei L., Su Y., Zhang D., Wang Y. (2021). Identifying the spatiotemporal dynamic of PM_2.5_ concentrations at multiple scales using geographically and temporally weighted regression model across China during 2015–2018. Sci. Total Environ..

[36] Fotheringham A.S., Brunsdon C., Charlton M. (2003). Geographically Weighted Regression: The Analysis of Spatially Varying Relationships.

[37] Zhou Q., Wang C., Fang S. (2019). Application of geographically weighted regression (GWR) in the analysis of the cause of haze pollution in China. Atmos. Pollut. Res..

[38] Wang S., Wang X., Lu F., Fan F. (2021). The impact of collaborative innovation on ecological efficiency—Empirical research based on China’s regions. Technol. Anal. Strateg. Manag..

[39] Wang S., Wang J., Fan F. (2021). The hidden mediating role of innovation efficiency in coordinating development of economy and ecological environment: Evidence from 283 Chinese cities. Environ. Sci. Pollut. Res..

[40] Fan F., Dai S., Zhang K., Ke H. (2021). Innovation agglomeration and urban hierarchy: Evidence from Chinese cities. Appl. Econ..

